# Resolution of Respect to Dr. Jonathan Taft

**Published:** 1903-12

**Authors:** 


### RESOLUTION OF RESPECT TO DR. JONATHAN TAFT.

WHEREAS, After a long and useful career of sixty years, as practitioner, author, journalist, and teacher, death has ended the life-work of Professor Jonathan Taft, who was universally loved and respected by the dental profession for his scholarly attainments and high ethical standing; and

WHEREAS, In the death of Dr. Taft our profession has lost an advanced thinker and an able and enthusiastic exponent of the best in dental surgery; therefore be it

*Resolved,* That the Fraternal Dental Society of St. Louis extend our sincere sympathy to Mrs. Taft in her bereavement, which is the bereavement of the whole profession, and express our high regard for the worth and character of this pioneer, who so ably exemplified the highest ideal of American dentistry.

Unanimously adopted, October 20, 1903.

W. L. WHIPPLE, *President pro tem.*
E. E. HAVERSTICK, *Secretary.*

